# Post COVID-19 treated with AstraZeneca vaccine exacerbates arthritis with susceptibility to herpes in an older woman: A case report

**DOI:** 10.1097/MD.0000000000048989

**Published:** 2026-05-22

**Authors:** David Calderon Guzman, Norma Osnaya Brizuela, Maribel Ortiz Herrera, Armando Valenzuela Peraza, Ernestina Hernandez García, Francisca Trujillo Jimenez, Hugo Juarez Olguin

**Affiliations:** a Laboratory of Neurosciences, Instituto Nacional de Pediatria (INP), Mexico City, Mexico; b Laboratory of Experimental Bacteriology, Instituto Nacional de Pediatria (INP), Mexico City, Mexico; c Laboratory of Pharmacology, Instituto Nacional de Pediatria (INP), Mexico City, Mexico; d Department of Pharmacology, Faculty of Medicine, Universidad Nacional Autonoma de Mexico, Mexico City, Mexico.

**Keywords:** arthritis, COVID-19, herpes, low back pain, sciatica

## Abstract

**Rationale:**

Rheumatoid arthritis (RA) is an autoimmune inflammatory disorder that has seriously affected human health worldwide. This report describes a rare case of herpes infection, secondary to RA, that emerged after coronavirus disease 2019 (COVID-19) infection or vaccination. Despite pharmacological intervention for arthritis, herpes, and mental health symptoms, the patient’s condition has not improved.

**Patient concerns:**

A 70-year-old female homemaker, weighing 110 lbs, sought urgent care for persistent dysphoric mood and irritability attributed to a post-COVID infection. The ill-health condition set in with severe pain, followed shortly by the onset of herpetic lesions. She experienced the symptoms while suffering from chronic fatigue syndrome.

**Diagnoses:**

A diagnosis of arthritis was made at the age of 60, and the patient has since been under treatment with analgesic therapy and prednisone. However, her doctor advised lowering the dose of her RA medications. At the age of 67, she developed herpes. She is currently on acyclovir and remains fully compliant with her medication. A simple skull magnetic resonance study depicted the absence of intracranial abnormality. Electromyography of normal muscles is silent at rest, without neuropathy. Cholesterol, sodium, chlorine, erythrocyte, and eosinophil were at the lower limit of normal, while lipase, phosphatase alkaline, and platelets were at the upper limit.

**Interventions:**

An integrative approach was taken, combining pharmaceutical prescriptions for arthritis, herpes, and mood symptoms with unconventional complementary therapies. Oral food intake was initiated and well-tolerated; however, the patient’s clinical condition did not improve with the current treatment. She is currently recovering at home on a regimen of vitamins, acyclovir, and analgesics. While she has shown clinical improvement, some complications have emerged.

**Outcomes:**

Diagnostic findings demonstrate that her health is not within normal limits, necessitating a daily regimen of prednisone, analgesic therapy, and hydroxyzine/losartan. This case highlights the importance of studying patients with RA. Risk factors for severe COVID-19 outcomes include older age and comorbidities.

**Lessons:**

This case highlights the potential for COVID-19 infection or immunization to precipitate a flare of preexisting arthritis, causing severe lower limb manifestations and sacroiliitis (low back pain/sciatica) in older individuals receiving management for rheumatic disease. Immune abnormalities in patients treated with prednisone are elements in deciding the prescription and duration of this drug, and a neurological assessment is essential.

## 1. Introduction

Immune homeostasis is a steady immune state that not only protects the host from pathogens but also prevents the emergence of pathological self-reactive immune cells. Rheumatoid arthritis (RA) is an autoimmune inflammatory disorder that has seriously affected human health worldwide. Alternative strategies for precise RA treatment include prednisone and dexamethasone.^[1]^ Furthermore, some biomarkers suggest that many membrane-attached, biologically active molecules – including tumor necrosis factor receptors (TNFRs) and the pro-inflammatorycytokine tumor necrosis factor-α – are cleaved by this enzyme. The 2 types of TNF receptors are TNFR1 and TNFR2. Inactive Rhomboid 2 belongs to the pseudo-protease class of the rhomboid family, and it is abundantly observed in the immune cells.^[2]^

Coronavirus disease 2019 (COVID-19) pandemic presents a great challenge in the management of patients with RA, who are generally more susceptible to infection events because of their autoimmune condition and the treatment with immunomodulatory drugs.^[3]^ The immunological response to the severe acute respiratory syndrome coronavirus 2 (SARS-CoV-2) virus and the treatment of COVID-19 disease present a potential risk for viral reactivation, particularly herpes simplex virus-1.^[4]^ The aim was to check if coronavirus disease or vaccines alter susceptibility to herpes infection in individuals with autoimmune conditions.

## 2. Methods

Assessment was completed and documented by the medical services of Neurology, Rheumatology, Cardiology, Psychology, and Ophthalmology.

This is a case of a 70-year-old female homemaker, weighing 110 lbs. She lives with her daughter in her own house. She sought urgent care for persistent dysphoric mood and irritability attributed to a post-COVID infection.

Clinical history: A diagnosis of arthritis was made at the age of 60, and the patient has since been under treatment with analgesic therapy and prednisone. At the age of 67, she developed herpes. She is currently on acyclovir and remains fully compliant with her medication. For some years now, she has been engaged in active walking, which she usually does 4 days per week. In all these years, she had been feeling completely stable when doing the exercises and carrying out her daily routines. Following a second COVID-19 infection, she continued her daily routine, reporting no lingering complications. The ill-health condition set in following a fall after a sudden wake-up from sleep. Since then, she has presented with severe lower limb manifestations, sacroiliitis (low back pain/sciatica), and a dysphoric mood with irritability, which usually flare at night.

Core tips: Patients with arthritis suffering from post-COVID-19 depression and anxiety may still require hospital visits for pain management, even when their condition does not remain stable despite medical treatment and nutritional support.

Medical history: RA at 60 years of age, hypertension since 65 years old, depapillation of tongue for 6 months, body pain for 2 weeks, weight loss 5%, herpes simplex since 67 years old, depression, and anxiety.

COVID-19 vaccination information: first dose (AstraZeneca), second dose (AstraZeneca), and third dose (AstraZeneca).

Arthritis managements: Prednisone 20 mg, twice daily. Dexamethasone 8 mg/2 mL as the need may be.

Hypertension managements: Losartan 50 mg, once daily. Nifedipine 30 mg, once daily.

COVID-19 symptom management: Beclometasone nasal spray (100 mcg). Administer as directed by a healthcare provider (typically twice daily). Paracetamol (500 mg): take up to the maximum daily allowance, as needed for pain. Amoxicillin (500 mg): twice daily.

Anxiety managements: Citalopram (20 mg): 1 time daily.

Depression managements: Hydroxyzine (10 mg): 1 time daily.

Analgesic managements: Celecoxib (200 mg): 3 times daily. Buprenorphine patch (5 mg): 1 time every 36 hours.

Herpes managements: Aciclovir (250 mg): twice daily. Herpes vaccines: first and second doses applied.

Diet: Chicken, pork, fish, eggs, beans rice, whole bread, corn, vegetables, fruits, celery and beetroot juice daily, lactose-free milk, sugar-free flavoring, olive oil. No-alcohol and no-smoking.

Review of systems: Waking 1+ times nightly to urinate.

Physical exam: Constitutional: the patient appears physically healthy and well-nourished, but exhibits noticeable irritability, consistent with reported anxiety and depression.

Vitals: Vital signs were recorded as follows: blood pressure and heart rate measured 130/91 mm Hg (107 bpm) while supine, 138/78 mm Hg (107 bpm) while sitting, and 146/95 mm Hg (115 bpm) while standing. The patient’s BMI is 20.0.

Head: The patient is normocephalic and atraumatic. Cardiac findings include tachycardia with a regular rhythm, but no murmurs, rubs, or gallops were noted.

Respiratory: Normal breath sounds heard in all fields.

EA: Exam reveals a soft, non-tender abdomen, free of distention or masses.

Musculoskeletal: Motor strength 5/5 bilaterally, including proximal (hip flexors) and distal muscle groups. Inflamed hand joints. Foot: No sign of callus, ulcer, or deformity found during foot inspection.

Neurology: Neurological assessment revealed normal cognitive function; motor examination showed no evidence of tremors, rigidity, or bradykinesia.

Cognitive screening: Assessment of sensation, proprioception, and motor function revealed abnormal deep tendon reflexes.

Identified fall risk factors: Assessment findings demonstrate reduced strength/balance, placing the patient at high risk for falls. She takes prednisone and an analgesic, along with daily hydroxyzine and losartan.

Fall prevention plans: She was advised to adjust or reduce her medication dosage for RA.

Complementary studies: Brain MRI (non-contrast) was normal.

Electromyography study: Normal muscles are silent at rest, without neuropathy in old patient.

Relevance of this case: The present case report highlights the potential for COVID-19 infection or immunization to precipitate a flare of preexisting arthritis, causing severe lower limb manifestations and sacroiliitis (low back pain/sciatica) in older individuals receiving management for rheumatic disease.

Ethical committee approval is not required for a single case arising from a standard practice. We used identifiable information and obtained a written consent from the participant’s relatives, as the patient had directly delegated decision-making to a family member – in this case, the daughter – who suggested that this could benefit patients with similar pathologies.

Table 1 shows all drug given to patient.

**Table 1:** Medication used in the old woman with arthritis post to COVID-19 pandemic.

Drugs	Doses
Sulfasalazine 500 mg	1 × 2 day
Methotrexate 2.5 mg	6 × week
Celecoxib 200 mg	2 × 2 day
Pregabalin 75 mg	2 × 2 day
Tramadol 37.5 mg + Paracetamol 325 mg	Pain as the need may be
Cytidine 5 – monophosphate 5 mg, Uridine 5-triphosphate 3 mg	1 × 2 day
Cannabis solution 125 mg/30 mL	20 drops night
Amitriptyline 25 mg	1 × 1 day
Primidone 250 mg	1 × 1 day
Hydroxyzine 10 mg	1 × 1 day
Alpha Lipoic Acid 600 mg	2 × 1 day
Complex B (B_1_-Thiamine, B_6_-Pyridoxine, B_12_-Cyanocobalamin)	1 × 1 day
Folic acid 5 mg	1 × 1 day
Perindopril 5 mg + Indapamida 1.25 mg	1 × 2 day
Losartan 50 mg	1 × 1 day
Nifedipine 30 mg	1 × 1 day
Hydrochlorothiazyde 10 mg	1 × 1 day
Vitamin D 100,000 U	1 × 1 day
Diclofenac 75 mg	Pain as the need may be

COVID-19 = coronavirus disease 2019.

All blood and serum test results (Tables 2–5) were generated by a College of American Pathologists accredited laboratory and are provided with relevant reference ranges.

**Table 2:** Blood analysis of sex woman old 70 years.

Assays	Values	Range
Glucose	94.0	82.0–115.0 mg/dL
Urea	24.7	16.6–48.5 mg/dL
Ureic nitrogen	11.5	8.0–23.0 mg/dL
Creatinine in serum	0.68	0.50–0.90 mg/dL
Uric acid in serum	3.5	2.4–5.7 mg/dL
Total cholesterol	163.6	≤200.0 mg/dL
Triglycerides	113.5	≤150.0 mg/dL
Cholesterol HDL	63.4*	≥65.0 mg/dL
Cholesterol LDL	81.0	≤100.0 mg/dL
Bilirubin total	0.44	0.00–1.20 mg/dL
Phosphatase alkaline (ALP)	115.3*	35.0–104.0 U/L
Calcium in serum	9.6	8.8–10.2 mg/dL
Phosphorus in serum	3.9	2.5–4.5 mg/dL
Magnesium in serum	2.3	1.6–2.4 mg/dL
Iron	115.2	33.0–193.0 μg/dL
Amilase	79.0	28.0–100.0 U/L
Lipase	64.80*	13.0–60.0 U/L
Sodium	125.7*	136.0–145.0 mmol/L
Potasium	4.7	3.5–5.1 mmol/L
Chloride	87.2*	98.0–107.0 mmol/L

Method: Photometry. Device: Cobas 8000 – Roche.

* Values near the upper or lower limit.

**Table 3:** Hematology analysis of sex woman old 70 years.

Assays	Values	Range
Leukocytes	5.05	3.98–10.04 (10^3^/μL)
Erythrocytes	3.52*	3.93–5.22 (10^6^/μL)
Hemoglobin	12.10	11.20–15.70 (g/dL)
Hematocrit	33.30*	34.10–44.90 (%)
Hemoglobin mean corpuscular	34.40*	25.60–32.20 (pg)
Platelets	395*	182–369 (10^3^/μL)
Platelets mean volume	8.90*	9.40–12.30 (fL)
Lymphocyte	29.5	19.3–51.7 (%)
Neutrophils	60.4	34.0–70.1 (%)
Monocytes	8.5	4.7–12.5 (%)
Eosinophils	0.4*	0.7–5.8 (%)
Basophil	0.6	0.1–1.2 (%)
Lymphocyte	1.49	1.18–3.74 (10^3^/μL)
Eosinophils	0.02*	0.04–0.36 (10^3^/μL)

Método: Flow Cytometry and SLS. Device: Sysmex XN-900.

* Values near the upper or lower limit.

**Table 4:** Urine analysis of sex woman old 70 years.

Assays	Value	Range
Color	Amber*	Yellow
Specific gravity	1.014*	1.016–1.022
pH	7.0	4.8–7.40
Esterase leukocyte	100/μL*	Negative (cel/μL)
Nitrites, proteins, glucose, cetones, bilirubin	Negative	Negative
Blood	25/μL*	Negative (cel/μL)

* Values near the upper or lower limit.

**Table 5:** Microscopic analysis of urine.

Assays	Value	Range
Leukocyte	1	0–2
Erythrocyte	4*	0–4
Bacteria	Moderate*	
Epithelial cells, crystals, yeasts, cylinders pathological, sperm	Negative	Negative

Method: Microscopy. Device: Cobas 6500 – Roche.

* Values near the upper or lower limit.

The results of blood and serum tests (Tables 2–5), were generated in a laboratory certified by College of American Pathologists, and the results are provided within standard reference intervals. Results are classified as normal if they fall within the provided reference interval values near or outside the limits are marked with (*), indicating high/low results, while any value exceeding these limits is considered abnormal. Cholesterol, sodium, chloride, erythrocytes, and eosinophils were at the lower limit of normal, while lipase, alkaline phosphatase, and platelets were at the upper limit. The clinical significance of urinalysis results may be attributed to hematuria or infection in the old patient.

## 3. Discussion

The key mechanism of critical illness development is the induction of immunosuppression via the dysfunction of interferon and interleukin pathways. The first-line checkpoint blockade treatment applied by clinicians for immune-related adverse events is the corticosteroids, specifically dexamethasone.^[5]^ Prednisone produces an immunosuppressive effect. By disrupting key iron metabolism proteins, the evaluated immunosuppressants may pose a greater risk to mineral metabolism than other treatments.^[6]^ Therefore, monitoring immune abnormalities in patients on prednisone is crucial for determining dosage and duration, a topic of interest to all rheumatologists.

In the present case, a post-COVID-19 patient with RA developed Herpes Zoster (Shingles) following pharmacotherapy. The risk of incident herpes is higher in RA patients, and it is further compounded by the prolonged use of steroids like prednisone.^[7]^ Veltman et al suggest that an autoimmune disease and prednisone may induce severe depression.^[8]^

Parenteral dexamethasone caused chronic herpes labialis reactivation in our patient. The immunosuppression induced by this long-acting corticosteroid likely triggered these reoccurrences by reducing vaccine-induced antigen-specific T-cell responses.^[9]^

RA is a common autoimmune disease characterized by chronic joint inflammation, leading to cartilage and bone erosion. While therapeutic intervention aims for disease remission, it can be hindered by poor structural recovery of the joints.^[10]^ In addition, medication-induced herpes reactivation can lead to unexpected neurological adverse events, such as acute transverse myelitis and acute demyelinating polyneuropathy.^[11]^ The above points explain the appearance of severe depression, neuralgia, lower back pain, and sciatica, among other conditions, in our elderly female patient with arthritis and post-COVID-19 syndrome.

In a recent study of 23 post–COVID-19 patients, 16 of whom were female, the authors found that 70% of female participants experienced inflammatory lower back pain. Clinical manifestations were varied, presenting primarily as asymmetric oligoarthritis of the lower limbs, with or without extra-articular features.^[12]^ However, the persistent reactivation of the herpesviridae family in immunocompromised patients may result in unfavorable outcomes for the hosts, a risk that rises exponentially with chronological age.^[13]^

The outbreak of coronavirus in the world has led to uncertainty regarding the right treatment for autoimmune patients since their immune system is weak and even exacerbated with the administration of immunosuppressive agents, thus predisposing them to a host of infections.^[14]^ COVID-19 can result in an extensive range of symptoms, such as taste dysfunction.^[15]^ This could explain why our patient with arthritis showed depapillation of the tongue for 6 months.

Current treatment options consist of various types of disease-modifying antirheumatic drugs; however, 20% to 30% of patients are partially resistant to them,^[16]^ probably due to age and tissue malfunction. In addition, current alternative therapies have limited efficacy in slowing disease progression and managing pain. This may be the reason why our patient had sought different conventional and unconventional treatment options, such as physical therapy, private and govern medicine, acupuncture, consumption of marijuana, herbal therapy to manage her severe pain, and ameliorate her depression and anxiety that impact her quality of life.

Physiotherapy (PT) and occupational therapy are non-pharmacological approaches that play a critical role in the management of RA patients through the alleviation of their pain and fatigue; hence, enhancing the overall quality of their life.^[17]^ Our patient received PT twice a week, and her pain showed slight improvement.

Acupuncture has been shown to have an immunosuppressive effect by restoring normal immune tolerance. Its mechanism is by augmenting NK and CD8+ T-cell function, and restoration of Th1/Th2, Th17/Treg, and M1/M2 balance.^[18]^ Our patient attended an acupuncture session but did not feel any improvement.

Studies on dietary influence on patients with RA have shown that poor dietary quality is linked with elevated levels of inflammation and pain.^[19]^ Nonetheless, our patient reports being obsessed with a healthy diet.

Cannabinoids possess beneficial properties as potential treatments for rheumatoid diseases due to their anti-inflammatory and analgesic properties. However, there are currently no established clinical recommendations regarding the utilization of cannabis for treating rheumatoid diseases.^[20]^ Our patient applied topical Cannabinoid-Based Medicines every night, supposedly to reduce joint and nerve pains.

Rhizome of turmeric (*Curcuma longa* L.) is a herbal plant with anti-inflammatory properties, which has been employed to abate many inflammatory conditions, such as arthritis, and to enhance the immune system.^[21]^ Our patient drinks Curcuma tea every morning.

Conversely, certain studies examine the association of nonconventional therapies with treatment noncompliance, specifically regarding the risks and benefits to which patients may be exposed by using complementary therapies.^[22]^

In fact, increasing knowledge of the pathophysiology of SARS-CoV-2 infection is turning the consideration ground to some antirheumatic drugs as potential treatments for the management of COVID-19.^[23]^ While the overall incidence of medication changes was similar before and after the American College of Rheumatology guidance for the management of rheumatic disease during the COVID-19 pandemic was first published in April 2020, physician-guided changes were more likely after publication.^[24]^

Since the appearance of the COVID-19 pandemic, it is assumed that vaccination reduces the risk of transmission to others; nevertheless, the results show that the viral load in the vaccinated population is not consistently lower compared to the unvaccinated. It is also reported that viral loads were mostly lower in reinfections compared to breakthrough infections. Indeed, there is no convincing evidence that the COVID-19 vaccination significantly reduces the risk of SARS-CoV-2 transmission,^[25]^ or that the number of COVID-19 cases is higher among the fully vaccinated population.

## 4. Conclusion

The present case demonstrates that COVID-19 infection or vaccination may activate or worsen arthritis, leading to low back pain and sciatica, particularly in old-aged patients with preexisting joint disease.

## Acknowledgments

We thank Dr Cyril Ndidi Nwoye Nnamezie, an expert translator and native English speaker, a researcher in Medical Science and a physician by profession. The authors express their profound gratitude to the Instituto Nacional de Pediatria (INP) for the support in the publication of this article issued on the Program A022.

## Author contributions

**Conceptualization:** David Calderon Guzman, Hugo Juarez Olguin.

**Formal analysis:** David Calderon Guzman, Maribel Ortiz Herrera, Hugo Juarez Olguin.

**Investigation:** David Calderon Guzman, Maribel Ortiz Herrera, Armando Valenzuela Peraza, Ernestina Hernandez García, Francisca Trujillo Jimenez, Hugo Juarez Olguin.

**Supervision:** David Calderon Guzman.

**Writing – original draft:** David Calderon Guzman, Hugo Juarez Olguin.

**Writing – review & editing:** David Calderon Guzman, Norma Osnaya Brizuela, Maribel Ortiz Herrera, Armando Valenzuela Peraza, Ernestina Hernandez García, Francisca Trujillo Jimenez, Hugo Juarez Olguin.

**Data curation:** Norma Osnaya Brizuela, Armando Valenzuela Peraza, Ernestina Hernandez García, Francisca Trujillo Jimenez.

**Methodology:** Norma Osnaya Brizuela, Armando Valenzuela Peraza, Ernestina Hernandez García, Francisca Trujillo Jimenez.

**Validation:** Norma Osnaya Brizuela, Maribel Ortiz Herrera, Armando Valenzuela Peraza.

**Software:** Maribel Ortiz Herrera, Armando Valenzuela Peraza, Francisca Trujillo Jimenez.

**Visualization:** Hugo Juarez Olguin.
